# A cancer-predisposing syndrome is always there: a sequential occurrence of acute lymphoblastic leukemia after acute myeloid leukemia in a child with down syndrome

**DOI:** 10.3389/fonc.2026.1937872

**Published:** 2026-09-24

**Authors:** Marta Arrabito, Nicolò Li Volsi, Emanuela Cannata, Manuela La Rosa, Luca Lo Nigro

**Affiliations:** 1 PhD program; Med-Clin Department; University of Catania, Catania, Italy; 2 Center of Pediatric Hematology Oncology Azienda Ospedaliero Universitaria Policlinico – San Marco, Catania, Italy; 3 Cytogenetic-Cytofluorimetric-Molecular Biology Lab, Center of Pediatric Hematology Oncology, Azienda Policlinico – San Marco, Catania, Italy

**Keywords:** acute lymphoblastic leukemia, acute myeloid leukemia, cancer predisposing syndromes, children, down syndrome

## Abstract

**Introduction:**

Down syndrome (DS) is the most common cancer predisposing syndrome (CPS) associated with a markedly increased risk of pediatric hematologic malignancies, specifically acute myeloid leukemia (AML) and acute lymphoblastic leukemia (ALL). Although both AML and ALL occur more frequently in children with DS than in the general pediatric population, the sequential development of both malignancies in the same patient is extremely rare.

**Results:**

Here we report the case of a child with DS who initially developed an AML and subsequentially an ALL, while the myeloid leukemia was in remission.

**Discussion:**

This case underscores the rarity of sequential AML and ALL in DS patients and supports the hypothesis that these leukemias represent biologically distinct diseases. Trisomy 21 has a central role in predisposing to both myeloid and lymphoid leukemogenesis through profound alterations of hematopoiesis and gene deregulation.

## Introduction

Down syndrome (DS), caused by full or partial trisomy of chromosome 21, is the most common chromosomal disorder, with an incidence of approximately one case in 1,000 live births (1). Due to the genetic, cellular, and molecular mechanisms associated with the gain of a third copy of chromosome 21 (2), children with DS are highly predisposed to develop hematologic malignancies, carrying a 10–20-fold higher risk of acute leukemia compared to the general pediatric population (3). Conversely, the frequency of solid tumors is significantly lower in individuals with DS (4).

Notably, DS patients exhibit a 150-fold increased risk of acute myeloid leukemia (AML) before the age of 5 years (5). Children with DS are frequently diagnosed with acute megakaryoblastic leukemia (AMKL), which is an uncommon subtype of AML classified as M7, according to the French–American–British (FAB) system (6). Myeloid leukemia associated with DS (ML–DS) results from a sequence of pathogenetic events leading to overt leukemia. Gain of chromosome 21 already perturbs *in utero* hematopoiesis, leading to erythroid predominance and a significant loss of B progenitors (7). This environment promotes the acquisition of driver mutations such as *GATA1*—a gene essential for the growth and maturation of erythroid and megakaryocytic cells (8, 9). These alterations could lead to transient abnormal myelopoiesis (TAM), a pre-leukemic state characterized by >10% myeloid blasts, which typically resolves spontaneously without chemotherapy (10). However, 10%–20% of neonates with TAM eventually develop ML-DS due to the acquisition of secondary mutations in transcriptional regulators and signaling pathways, including cohesin complex genes, JAK family kinases, and epigenetic regulators (11, 12). A very recent article reveals that cohesin and PRC2 insufficiencies converge on PRC2 dysfunction while exerting distinct epigenetic effects and synergize with trisomy 21 and GATA1s to remodel the epigenetic landscape, driving progression from a preleukemic state to overt leukemia (13).

Furthermore, children with DS have a 20-fold increased risk of developing acute lymphoblastic leukemia (ALL) (14) almost exclusively of B-cell phenotype (15). Approximately 50% of patients overexpress *CRLF2* and 20% present *JAK2* mutations (16).

Although the individual frequencies of AML and ALL are considerably high in children with DS, their sequential occurrence in the same patient is exceptional. Thus, we report the case of a child with DS who developed AML and, 4 years later, ALL while in complete remission from the first malignancy. Precisely, our patient did not present any transient abnormal myelopoiesis at birth.

## Case description

A 1-year-old female with DS was diagnosed with M7 AML (according to FAB classification). The immunophenotype analysis identified blasts positive for CD45/CD33/CD7/CD41a/CD61/CD117/CD56, and the karyotype was *47,XX,t(5;12)(q13;p11.2),+21c[23]/47,XX,+21c[5].* She was treated according to AML 2002/02 protocol and achieved complete remission (CR) (**Table 1**).

**Table 1:** Main clinical, laboratory, and biologic features of the patient during AML and ALL.

	Acute myeloid leukemia	Acute lymphoblastic leukemia
Age	15 months	5 years, 10 months
FAB	M7	L1
Immunophenotype	Pos: CD45/CD33/CD7/CD41a/CD61/CD117/CD56; Neg: MPO/CD13/CD34/lymphoid	Pos: CD10/CD19/CD79a/DR/CD22/CD34/CD117; Neg: CD66c/CD9/myeloid
Karyotype	47,XX,t(5;12)(q13;p11.2),+21c[23]/47,XX,+21c[5]	47,XX,+21[8]
Molecular screening	Negative	*IKZF*wt, *EBF1*wt, *PAX5*wt, *ETV6*wt, *RB1-26*wt, *JAK2*wt, *CDKN2A*wt, *CRLF2*wt
Stratification risk	SR	HR
Treatment	Protocol AIEOP-2002/02	Protocol AIEOP-BFM 2009
Outcome	Alive in 1st CR	Alive in 1st CR

FAB, French–American–British; Pos, positive; Neg, negative; wt, wild type; SR, standard risk; HR, high risk; CR, complete remission.

After 4 years of follow-up, she was diagnosed with a B-lineage ALL and treated according to AIEOP ALL 2009 protocol (17). The immunophenotype analysis detected blasts expressing CD10/CD19/CD79a/DR/CD22/CD34/CD117. Molecular analyses were negative for *IKZF*, *EBF1*, *PAX5*, *ETV6*, *RB1-26*, *JAK2*, *CDKN2A*, and *CRLF2*. There was no central nervous system (CNS) involvement. Based on the bone marrow blasts percentage on day 15 of treatment, she was assigned to the high risk (HR) group (**Table 1**). During consolidation therapy, the patient developed right pulmonary artery thrombosis, which required heparin. Moreover, during the reinduction phase, persistent cough associated to neutropenia prompted a chest CT, which revealed a large air-filled cavitation in the left lung with predominantly parenchymal consolidation. Bronchoscopy with bronchoalveolar lavage (BAL) was positive for rhinovirus and herpes simplex virus (HSV-1). Because of clinical deterioration, a subsequent CT showed increased cavitation in the left lung, mediastinal shift, and a left pneumothorax, which required chest tube placement. Despite the prompt intervention, the child was transferred to the intensive care unit (ICU) for 20 days. After resolution of the pulmonary complications, she resumed and completed therapy 2 years after the ALL diagnosis. Currently, at 14 years old, she remains in good clinical condition and in complete remission from both AML and ALL.

## Discussion

Our patient represents a rare case of a sequential occurrence of both AML and ALL in DS. Currently, few similar cases were described in the literature, as reported in **Table 2**. Tomizawa et al. reported a case series of four DS patients who firstly developed AML and later ALL (18). They estimated a frequency of DS children experiencing both malignancies at approximately 0.9% (18). All patients presented with an M7 phenotype and were 1 to 2 years old at onset, and TAM occurred previously in two of them (18). A minimally intensive DS AML-oriented chemotherapy was administered, allowing complete remission. Age at onset of ALL ranged from 7 to 15 years, and all children showed a B cell precursor (BCP) phenotype, without CNS involvement. None of the four cases presented cytogenetic anomalies generally found in non-DS pediatric ALL (18). Hellebostad et al. described a child with DS who developed presumed TAM, followed by AML at age of 3 years, and then presented with ALL when she turned 6 years. A *GATA1* mutation was detected in the AML but not in ALL clone, highlighting that there were two different diseases (19). Murphy et al. reported a Hispanic female with DS diagnosed with AMKL at the age of 17 months, who remained in remission until 7 years old when B-cell ALL occurred: she is currently in remission (20). They also performed a Children’s Oncology Group (COG) retrospective review and revealed five patients with DS manifesting with ALL following AML. The age at onset of AML ranged from 1 to 32 months, with a mean of 15 months. Age at ALL presentation ranged from 2 to 12.6 years, with a mean of 5.3 years, and all patients were alive. They calculated the frequency of developing ALL after AML to be 0.58% (20).

**Table 2:** Cases with DS and AML followed by ALL, reported in literature.

	Number of patients	Sex	Age—AML onset	Age—ALL onset	TAM	Incidence	Outcome
Hellebostad et al. (2005) (19)	1	Female	3 years old	6 years old	Yes	na	Remission for 2 years
Tomizawa et al. (2017) (18)	4	3 female, 1 male	Range: 1 to 2 years	Range: 7 to 15 years	2 yes, 2 no	0.9%	3 in CCR, 1 dead
Murphy et al. (2019) (20)	6	3 female, 3 male	Range: 1 to 32 months	Range: 2 to 12.6 years	1 no, 5 na	0.58%	CCR with an average follow-up of 10 years

TAM, transient abnormal myelopoiesis; na, not available; CCR, complete continuous remission.

Accordingly, our patient presented M7 AML at the age of 1 year old and the B cell precursor ALL 4 years later. In line with other reported cases, it seems that there was no relationship between AML therapy administered and the development of ALL. Therapy-related leukemia is a well-studied phenomenon described since 1970, with the most common malignancy identified as AML at an incidence of 2%–10% (21). However, our child did not present biological characteristics of secondary malignancies caused by prior cancers or cancer therapies, and different cytogenetic factors between AML and ALL have led to the occurrence of two separate malignancies, likely caused by a genetic predisposition to acute leukemias. Thus, the possible explanation is related to the presence of a third chromosome 21. Recent evidence suggests that this additional chromosome is completely responsible for the hematologic abnormalities and encodes genes causing ML-DS and DS-ALL leukemogenesis (22, 23). Trisomy 21 contributes to alter fetal hematopoiesis through genome-wide transcriptional perturbation, encompassing genes encoding transcription factors, pro-inflammatory cytokines, and several microRNA in fetal hematopoietic stem cells and in stromal cells (24). Furthermore, Crispino et al. detected key chromosome 21 genes (*ERG*, *CHAF1B*, and *DYRK1A*) that could be responsible for the higher risk of ML-DS in children with DS (25).

Our patient completed the AML protocol without any complication. Children with ML-DS indeed have a very favorable prognosis, reaching an event-free survival of approximately 90% thanks to the blasts’ hypersensitivity to chemotherapy (26). In contrast, children with DS–ALL have an inferior outcome and more chemotherapy-related toxicities compared with non-DS children (27), and this might be associated with the relative resistance of their lymphoblasts to cytotoxic drugs. Conversely, myeloblasts from patients with ML-DS showed higher susceptibility to cytotoxic drugs, contributing to increased cure rates (28). Toxicities frequently reported include cardiotoxicity, myelosuppression, lung infections, and mucositis, as described in our case (29, 30). The explanation for the high rates of chemotherapy-related toxicities in these patients is multifactorial. It could be related to impaired pharmacokinetic parameters that can lead to the toxicity of certain drugs such as methotrexate (31). Furthermore, some non-immunologic factors may also contribute to the increased risk of infections, such as pneumonia and sepsis: congenital heart disease, anatomical abnormalities of the airways, congenital anomalies of the lower respiratory tract, and gastroesophageal reflux disorders (32).

DS children with leukemia represent a special population that need specific biological studies and risk-adapted treatment protocols. The occurrence of both AML and ALL is very rare in these patients, and a larger pediatric cohort is necessary to better define disease mechanisms, treatment, and clinical outcome. Moreover, we would highlight that because of a heightened risk for late relapse or refractory disease, we have to implement lifelong, individualized follow-up protocols and dynamic risk stratification management for pediatric patients with DS-associated hematological malignancies.

## Data Availability

The raw data supporting the conclusions of this article will be made available by the authors, without undue reservation.

## References

[1] Bull MJ . Down syndrome. N Engl J Med. (2020) 382:2344–52. doi: 10.1056/nejmra1706537 32521135

[2] Sato T Yoshida K Toki T Kanezaki R Terui K Saiki R . Landscape of driver mutations and their clinical effects on Down syndrome–related myeloid neoplasms. Blood. (2024) 143:2627–43. doi: 10.1182/blood.2023022247 38513239

[3] Hasle H Clemmensen IH Mikkelsen M . Risks of leukaemia and solid tumours in individuals with Down’s syndrome. Lancet. (2000) 355:165–9. doi: 10.1016/s0140-6736(99)05264-2 10675114

[4] Osuna-Marco MP López-Barahona M López-Ibor B Tejera ÁChecktae . Ten reasons why people with Down syndrome are protected from the development of most solid tumors -A review. Front Genet. (2021) 12:749480. doi: 10.3389/fgene.2021.749480 34804119 PMC8602698

[5] Lange BJ Kobrinsky N Barnard DR Arthur DC Buckley JD Howells WB . Distinctive demography, biology, and outcome of acute myeloid leukemia and myelodysplastic syndrome in children with Down syndrome: Children’s Cancer Group Studies 2861 and 2891. Blood. (1998) 91:608–15. 9427716

[6] Arber DA Orazi A Hasserjian R Thiele J Borowitz MJ Le Beau MM . The 2016 revision to the World Health Organization classification of myeloid neoplasms and acute leukemia. Blood. (2016) 127:2391–405. doi: 10.1182/blood-2016-03-643544 27069254

[7] Jardine L Webb S Goh I Quiroga Londoño M Reynolds G Mather M . Blood and immune development in human fetal bone marrow and Down syndrome. Nature. (2021) 598:327–31. doi: 10.1038/s41586-021-03929-x 34588693 PMC7612688

[8] Grimm J Heckl D Klusmann J-H . Molecular mechanisms of the genetic predisposition to acute megakaryoblastic leukemia in infants with Down syndrome. Front Oncol. (2021) 11:636633. doi: 10.3389/fonc.2021.636633 33777792 PMC7992977

[9] Kosmidou A Tragiannidis A Gavriilaki E . Myeloid leukemia of Down syndrome. Cancers. (2023) 15:3265. doi: 10.3390/cancers15133265 37444375 PMC10340042

[10] Bhatnagar N Nizery L Tunstall O Vyas P Roberts I . Transient abnormal myelopoiesis and AML in Down syndrome: an update. Curr Hematol Malig Rep. (2016) 11:333–41. doi: 10.1007/s11899-016-0338-x 27510823 PMC5031718

[11] Labuhn M Perkins K Matzk S Varghese L Garnett C Papaemmanuil E . Mechanisms of progression of myeloid preleukemia to transformed myeloid leukemia in children with Down syndrome. Cancer Cell. (2019) 36:123–138.e10. doi: 10.1016/j.ccell.2019.08.014 31303423 PMC6863161

[12] Nikolaev SI Santoni F Vannier A Falconnet E Giarin E Basso G . Exome sequencing identifies putative drivers of progression of transient myeloproliferative disorder to AMKL in infants with Down syndrome. Blood. (2013) 122:554–61. doi: 10.1182/blood-2013-03-491936 23733339

[13] Suzuki Y Nakajima-Takagi Y Oshima M Ochi Y Saiki-Tsuchiya A Koide S . Polycomb repressive complex 2 insufficiency underlies myeloid leukemia in Down syndrome. Blood. (2026) 148:462–76. doi: 10.1182/blood.2025032083 41949996

[14] Izraeli S Vora A Zwaan CM Whitlock J . How I treat ALL in Down’s syndrome: pathobiology and management. Blood. (2014) 123:35–40. doi: 10.1182/blood-2013-07-453480 24235135

[15] Buitenkamp TD Izraeli S Zimmermann M Forestier E Heerema NA Van Den Heuvel-Eibrink MM . Acute lymphoblastic leukemia in children with Down syndrome: a retrospective analysis from the Ponte di Legno study group. Blood. (2014) 123:70–7. doi: 10.1182/blood-2013-06-509463 24222333 PMC3879907

[16] Nikolaev SI Garieri M Santoni F Falconnet E Ribaux P Guipponi M . Frequent cases of RAS-mutated Down syndrome acute lymphoblastic leukaemia lack JAK2 mutations. Nat Commun. (2014) 5:4654. doi: 10.1038/ncomms5654 25105841 PMC4665216

[17] Gottschalk H Möricke A Conter V Schrappe M Starý J Cario G . Reducing daunorubicin in induction therapy in children with B-lineage ALL with favorable prognosis: Results of phase III trial AIEOP-BFM ALL 2009. J Clin Oncol. (2025) 43:3739–49. doi: 10.1200/jco-25-01357 41213101

[18] Tomizawa D Endo A Kajiwara M Sakaguchi H Matsumoto K Kaneda M . Acute lymphoblastic leukemia in patients with Down syndrome with a previous history of acute myeloid leukemia. Pediatr Blood Cancer. (2017) 64:e26411. doi: 10.1002/pbc.26411 28012245

[19] Hellebostad M Carpenter E Hasle H Mitchell C Vyas P . GATA1 mutation analysis demonstrates two distinct primary leukemias in a child with Down syndrome; implications for leukemogenesis. J Pediatr Hematol/Oncol. (2005) 27:408–9. doi: 10.1097/01.mph.0000172223.04694.c4 16012335

[20] Murphy BR Roth M Kolb EA Alonzo T Gerbing R Wells RJ . Development of acute lymphoblastic leukemia following treatment for acute myeloid leukemia in children with Down syndrome: a case report and retrospective review of Children’s Oncology Group acute myeloid leukemia trials. Pediatr Blood Cancer. (2019) 66:e27700. doi: 10.1542/peds.18.4.643 PMC6941434 30908863

[21] Michels SD McKenna RW Arthur DC Brunning RD . Therapy-related acute myeloid leukemia and myelodysplastic syndrome: a clinical and morphologic study of 65 cases. Blood. (1985) 65:1364–72. doi: 10.1182/blood.v65.6.1364.bloodjournal6561364 3857944

[22] Roberts I Izraeli S . Haematopoietic development and leukaemia in Down syndrome. Br J Haematol. (2014) 167:587–99. doi: 10.24287/1726-1708-2015-14-3-13-33 25155832

[23] Laurent AP Kotecha RS Malinge S . Gain of chromosome 21 in hematological Malignancies: lessons from studying leukemia in children with Down syndrome. Leukemia. (2020) 34:1984–99. doi: 10.1038/s41375-020-0854-5 32433508 PMC7387246

[24] Baruchel A Bourquin J-P Crispino J Cuartero S Hasle H Hitzler J . Down syndrome and leukemia: from basic mechanisms to clinical advances. Haematol. (2023) 108:2570–81. doi: 10.3324/haematol.2023.283225 37439336 PMC10542835

[25] Ling T Zhang K Yang J Gurbuxani S Crispino JD . Gata1s mutant mice display persistent defects in the erythroid lineage. Blood Adv. (2023) 7:3253–64. doi: 10.1182/bloodadvances.2022008124 36350717 PMC10336263

[26] Barwe SP Kolb EA Gopalakrishnapillai A . Down syndrome and leukemia: An insight into the disease biology and current treatment options. Blood Rev. (2024) 64:101154. doi: 10.1016/j.blre.2023.101154 38016838

[27] Salazar EG Li Y Fisher BT Rheingold SR Fitzgerald J Seif AE . Supportive care utilization and treatment toxicity in children with Down syndrome and acute lymphoid leukaemia at free‐standing paediatric hospitals in the United States. Br J Haematol. (2016) 174:591–9. doi: 10.1111/bjh.14085 27161549 PMC5684439

[28] Zwaan CM . Different drug sensitivity profiles of acute myeloid and lymphoblastic leukemia and normal peripheral blood mononuclear cells in children with and without Down syndrome. Blood. (2002) 99:245–51. doi: 10.1182/blood.v99.1.245 11756178

[29] Palle J Frost B-M Peterson C Gustafsson G Hellebostad M Kanerva J . Doxorubicin pharmacokinetics is correlated to the effect of induction therapy in children with acute myeloid leukemia. Anti-Cancer Drugs. (2006) 17:385–92. doi: 10.1097/01.cad.0000198911.98442.16 16549995

[30] Hefti E Blanco JG . Anthracycline-related cardiotoxicity in patients with acute myeloid leukemia and Down syndrome: a literature review. Cardiovasc Toxicol. (2016) 16:5–13. doi: 10.1007/s12012-015-9307-1 25616318 PMC4514565

[31] Hefti E Blanco JG . Pharmacokinetics of chemotherapeutic drugs in pediatric patients with Down syndrome and leukemia. J Pediatr Hematol/Oncol. (2016) 38:283–7. doi: 10.1097/mph.0000000000000540 26907658 PMC4842084

[32] Hassler A Bochennek K Gilfert J Perner C Schöning S Creutzig U . Infectious complications in children with acute myeloid leukemia and Down syndrome: analysis of the prospective multicenter trial AML‐BFM 2004. Pediatr Blood Cancer. (2016) 63:1070–4. doi: 10.1002/pbc.25917 26814618

